# Circadian Organelles: Rhythms at All Scales

**DOI:** 10.3390/cells10092447

**Published:** 2021-09-16

**Authors:** Rona Aviram, Yaarit Adamovich, Gad Asher

**Affiliations:** Department of Biomolecular Sciences, Weizmann Institute of Science, Rehovot 7610001, Israel; rona.aviram@weizmann.ac.il (R.A.); yaarit.adamovich@weizmann.ac.il (Y.A.)

**Keywords:** circadian clocks, rhythms, organelles, mitochondria, nucleus, endoplasmic reticulum, lysosomes

## Abstract

Circadian clocks have evolved in most light-sensitive organisms, from unicellular organisms to mammals. Consequently, a myriad of biological functions exhibits circadian rhythmicity, from behavior to physiology, through tissue and cellular functions to subcellular processes. Circadian rhythms in intracellular organelles are an emerging and exciting research arena. We summarize herein the current literature for rhythmicity in major intracellular organelles in mammals. These include changes in the morphology, content, and functions of different intracellular organelles. While these data highlight the presence of rhythmicity in these organelles, a gap remains in our knowledge regarding the underlying molecular mechanisms and their functional significance. Finally, we discuss the importance and challenges faced by spatio-temporal studies on these organelles and speculate on the presence of oscillators in organelles and their potential mode of communication. As circadian biology has been and continues to be studied throughout temporal and spatial axes, circadian organelles appear to be the next frontier.

## 1. Introduction

“Clocks, processes measuring absolute time, occur in living organisms”, wrote Colin Pittendrigh in 1954 [1]. Considered one of the founding fathers of the clock field, Pittendrigh was among the first to deliberately adopt the term “clock”. This was done in contemplation of the growing body of evidence for daily rhythmic behaviors in various organisms [2]. To name a few: leaf movement in plants, bird navigation, and fly emergence from puparia, all had clear rhythms with daily periodicity. While some researchers initially objected to the concept of an internal clock and advocated for an (unknown) external factor that regulates these rhythms, most readily adopted the current view that light-sensitive organisms have an internal capacity to tell time.

In addition to internal timekeeping capacities, adopting the clock terminology instigated basic questions regarding its nature and clock-like properties [3]. For one, since a clock is a physical object and therefore must have a distinct location, an important question emerges: where is this biological clock located? This question has been echoed throughout decades in the search for, and subsequent discovery of, the location of the clock in a number of model organisms. In the 1970s, seminal lesion experiments in rodents revealed that the “master clock” in mammals resides in the Suprachiasmatic Nucleus (SCN) region of the brain [4,5]. Years later, clocks were found in almost every cell throughout the body [6,7,8,9] and were shown to function in a self-sustained and cell-autonomous manner in culture. Importantly, this latter finding goes together with the presence of circadian clocks in unicellular organisms, such as cyanobacteria [10].

**Figure 1:**
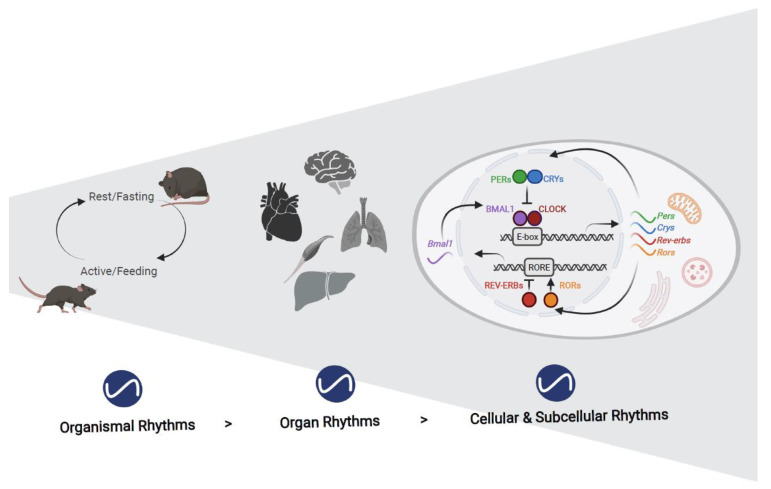
Circadian rhythmicity across multiple levels. Circadian rhythmicity occurs in myriad of biological functions, from behavior to physiology at the whole animal level and through specific tissue functions and cellular functions to subcellular processes. The circadian clock functions at the molecular level based on transcription-translation feedback loops.

Another fundamental implementation of the clock terminology is that a clock has parts, and consequently, chronobiologists were ever-eager to answer the question: what is the biological clock made of? The first to be identified of what are now termed “clock genes” was the *period* gene in *Drosophila melanogaster*, as its mutated forms were found to disrupt behavioral rhythms [11]. Intensive cloning efforts and genetic investigations enabled, by 1990, the introduction of a negative transcription and translation feedback loop (TTFL) model to explain how these genes and their products generate self-sustained oscillations [12,13,14]. Subsequent studies identified similar TTFLs consisting of additional clock genes in other organisms. Consequently, the TTFL is widely considered as a universal clock mechanism [15]. In mammals, the Circadian Locomotor Output Cycles Kaput (CLOCK) was the first clock component to be identified [16,17]. Successive studies further deciphered the mammalian clockwork, which consists of an intricate network of molecular feedback loops. The rhythmic heterodimerization of BMAL1 and CLOCK (or its paralog NPAS2) drives the expression of *Period* (*Per1*, *Per2*, and *Per3*) and *Cryptochrome* (*Cry1* and *Cry2*) genes. In turn, PERIOD and CRYPTOCHROME proteins accumulate and repress the transcription of their own genes. Another central feedback loop involves the expression of the nuclear receptors NR1D1/2 (REV-ERBα/β) and ROR, which are targets of BMAL1 transcription and regulate *Bmal1* expression [18,19] (Figure 1). Throughout the years, additional transcription regulators have been identified to participate in the transcriptional feedback loops (e.g., DEC1 and DEC2 [20,21]) and control rhythmic gene expression (e.g., the PARbZip transcription factors [22]). It is noteworthy that several studies also reported on circadian rhythmicity that is independent of a functional TTFL, for example [23,24].

The term *circadian* was coined as a conjugation of the Latin words *circa* (about) and *diem* (day) [25]. This was due to the fact that the actual period length of these clocks is close but seldom identical to 24 h. This frequency can be longer or shorter than a day, depending on the organism or environmental conditions, but for any given context, it would always be very stable. Importantly, the deviation from 24 h is only observable under what is called “free running conditions”, i.e., in constant routine experiments. The clock can continue to tick on its own, with a fixed period, highlighting its autonomous, internal nature. Any process which continues to cycle, with one major peak in ~24 h, in a constant environmental setup, is said to be under circadian clock control, or simply, circadian. Alternatively, a rhythmic phenomenon that repeats itself, but in a rhythmic environment such as the normal light/dark cycles would be called a “daily” or “diurnal” rhythm, as the rhythms might occur merely in response to the external cues. Such a rhythm can ultimately be deemed circadian, but only if it maintains its oscillations in free running settings. Often, the terms circadian and daily are unfortunately misused in the literature without adhering to their true definition.

Another circadian property is the capacity of adjusting to external time, also termed phase resetting, or entrainment. Many environmental signals can serve as timing cues, or zeitgebers (*time givers*, in German); prominent examples amongst them are light/dark, feeding/fasting, and temperature cycles [26,27,28]. Molecular input signals include, among others: glucocorticoids, cAMP, growth factors [29], glucose [30], insulin [31], and gases such as oxygen [32] and carbon dioxide [33].

From an evolutionary perspective, the presence of circadian clocks from unicellular organisms to plants, flies, and mammals suggests that the circadian clock confers survival advantages. For one, it can allow for temporal separation of conflicting processes and anticipation of the cycling environmental conditions (e.g., coordination between metabolic gene expression and activity with feeding–fasting rhythms). Thus, countless biological functions are rhythmic at multiple scales, from behavior to physiology and various cellular functions, even in subcellular compartments.

In this review, we summarize the evidence for ~24 h rhythmicity in the following membrane-bound organelles: nuclei, mitochondria, Endoplasmic Reticulum (ER), and lysosomes, as the majority of the literature relates to them. However, there have been some sporadic reports on other organelles, such as the Golgi network [34] or peroxisomes [35]. Notably, the lion’s share of these studies was performed in rodents and predominantly the liver, which is highly rhythmic [36]. We concentrated on the organelles’ structure and function, as could be gleaned by their morphology and key biochemical processes. We also discuss the importance, alongside the challenges, of studying the rhythmicity of intracellular organelles, as well as the potential implications of what we know so far. We hope this will leave the reader with a comprehensive view and serve as “food for thought” for future studies along this line of research.

## 2. Rhythms in Organelles

### 2.1. Nucleus

Polyploidy, an increase in the number of chromosome sets per cell, is a common feature of the mammalian liver, with a range of 30–90% polyploid hepatocytes in different organisms [37,38]. Polyploidy prevalence is generally associated with aging, senescence, and pathological conditions [38]. Nevertheless, the process is considered reversible, and daily rhythmicity in polyploidy was shown in mouse liver using both histological slices and flow cytometry of isolated nuclei [39]. For instance, bi-nucleated diploid hepatocytes peaked during the light phase, while mono-nucleated tetraploid hepatocytes peaked in the dark. Along these lines, Chao and colleagues observed a marked increase in hepatic polyploidy in clock-disrupted *Per1*,*2*,*3* triple negative mice and therefore hypothesized this to be clock-controlled [40]. However, as these experiments were carried out only at a single time of the day, it remains unclear whether the circadian clock controls temporal dynamics in ploidy.

Within the nuclei, DNA is folded into 3D structures, which are non-random and define spatial positioning between regulatory elements and coding regions [41,42]. The overall spread of chromatin spans throughout the nucleus, from areas which are centrally located and rich in transcriptionally active genes (called “compartment A”) as opposed to areas more peripherally localized with the nuclear lamina with repressed genes (“compartment B”). Within these “megastructures”, anchored by cohesin and CCCTC-binding factor (CTCF) proteins, are Topologically Associating Domains (TADs), which are mostly conserved and stable between different tissues and cell-states. At a small genomic scale, the spatial arrangement of genes and their regulating elements, namely, proximity between enhancers and promoters, was found to be very dynamic, and the chronobiology field was not late to the game. 

The first attempts to unravel daily chromatin rhythmicity were made in cell cultures. Using chromosome conformation capture-on-chip (4C) on the *Dbp* gene in Mouse Embryonic Fibroblasts (MEFs), [43] circadian rhythms in chromatin changes were detected. These rhythms in chromatin association appeared altered in clock-disrupted MEFs, though due to the low temporal resolution, the conclusions in this respect were limited. In the human colon cancer cell line HCT116, [44] rhythmic repressive Lamina Associated Domains (LADs) were shown following the interaction between PARP1 (Poly (ADP-Ribose) Polymerase 1), a chromatin modifier and integrator of feeding with the circadian clock [45], and CTCF. Evidence for interaction between the nuclear periphery and circadian rhythmicity had also been obtained in vivo, as levels of the nuclear envelope protein LaminA were shown to affect the rhythmicity of PER2 protein in mouse liver, and genetic manipulation of additional nuclear envelope-related proteins affected the period of behavioral rhythms in constant dark [46,47]. In addition, daily changes in open and closed configurations of chromatin compartments A and B were identified using Principal Component Analysis (PCA) analysis on Hi-C data [48].

While the functional significance of some of the long-range interactions remains debatable [41], and not all initial findings were recapitulated in vivo [49], a stream of studies has since underscored the importance of temporal examination of chromatin architecture, at least on the smaller genomic scale [41,42,50]. Circadian chromosome dynamics have been shown to involve clock components such as *Cry1* in mouse liver, which is controlled by *Rev-erbα* [51] and *Bmal1* [52]. Rhythmic enhancer activity of *Bmal1* and *Period2* was associated with transcription of these genes [53]. These clock genes, among others, present daily changes in their promoter contact sites [48]. In addition, several clock output genes undergo rhythmic cis element binding, such as: *Gys2* [52] and *Mreg* [53]. 

Though further studies are expected to produce a more complete picture, compartmentalization of the nuclear interior is by now established as a mechanism for generating diurnal rhythms in gene expression [41,42,50]. Gene transcription, which occurs in the nucleus, is the molecular basis for circadian clock function and control of its many rhythmic outputs. This notion, together with the rapid advancement in technologies for gene expression analyses in recent years, has positioned transcriptomic as one of the most prominent techniques in the chronobiologist toolkit. Over the past three decades, gene expression analyses have been fertile grounds for circadian studies and suggest that up to 40% of the genes in mammals are transcribed in a circadian manner [42,54,55]. Not only do transcription factor bindings cycle in a daily manner, but rhythms are also prevalent throughout major steps of transcription regulation and RNA processing such as chromatin accessibility, PolII activity, RNA capping, splicing, and export to cytoplasm [56].

Hence, gene transcription, which is a hallmark of nuclear function, is clearly rhythmic. Moreover, it carries wide implications on the rhythmicity of other cellular organelles as detailed below. That said, rhythmic transcription is not the end of the story, and as a matter of fact, not all rhythmic transcripts yield rhythmic protein levels [57,58]. Several studies in recent years applied proteomics and phospho-proteomics on whole cells as well as nuclear fractions isolated from mouse liver around the clock [39,57,58,59,60]. Under a rhythmic environment (light/dark and feeding/fasting cycles), the nuclear proteome undergoes significant diurnal changes with night-time enrichment for protein complexes involved in cytoskeleton organization, protein transport, proteolysis, and chaperoning of proteins [39]. Comparison of total and nuclear liver proteome shows a discrepancy between the two datasets, which implies that protein translocation from the cytoplasm into the nucleus plays a role in nuclear protein rhythmicity. In support of that, UniProt annotation of the nucleus-rhythmic proteins shows many are shuttling-related. In this respect, it is interesting to note that specific clock components themselves undergo rhythmic import into the nucleus, e.g., PER1 via Transportin 1 [61] as well as PERs and CRYs via NRON complex [62] and via KPNB1 [63].

Aside from transcriptomics and proteomics, circadian metabolomics have been at the forefront in recent years. In the last decade, following major technological advances, circadian lipid molecules were discovered across many cell types as blood, saliva, muscle cells and whole liver [64,65,66,67]. Following biochemical fractionations for the nucleus and mitochondria from mouse liver, lipids were found to undergo vast daily changes in these compartments [68]. In the nucleus, over 30% of the lipids are rhythmic, with higher levels in the beginning of the light phase. Their rhythmicity and overall abundance greatly respond to the feeding regimen. Interestingly, the nuclear lipid phases mirror those in mitochondria and suggest coupling and potential daily lipid shuttling between these organelles, which is further discussed below.

Taken together, the above detailed findings suggest temporal compartmentalization of key basic biological functions of the nucleus.

### 2.2. Mitochondria

Mitochondria have been of major interest for chronobiologists, and several aspects of circadian mitochondrial biology have been elucidated. Generally, there are three main facets through which the circadian clock exerts its control over mitochondrial function: (1) mitochondrial dynamics, namely cycles of fission and fusion to maintain shape, size, and number; (2) molecular content, namely expression of mitochondrial related genes (both nuclear and mitochondrial encoded) as well as proteins (e.g., enzymes) and metabolite levels; and (3) major mitochondrial functions such as oxidative phosphorylation, probed mostly through examination of Oxygen Consumption Rate (OCR). These three broad and interconnected categories (dynamics, content, and function) will serve here to navigate through the mitochondrial circadian literature [69,70].

Dynamic changes in morphology, from fragmented to tubular forms, contribute to mitochondrial functionality and involve continuous fission and fusion cycles to maintain their abundance, morphology, quality, and function [71]. Specifically, mitochondrial fission results in small and round mitochondria, while mitochondrial fusion generates thin and elongated mitochondria with highly interconnected networks.

Upon nutrient availability, mitochondria are mostly fragmented, and when nutrients are scarce, mitochondria become tubular and thus more supportive of ATP production [70,72]. Therefore, mitochondrial dynamics likely respond to daily rest/activity and feeding/fasting cycles due to differences in nutrient availability and energy demands. Indeed, early observations made on rat hepatocytes using electron microscopy reported significant differences in the volume and shape of mitochondria between rest and active phases [73]. Studies in mouse liver showed that mitochondrial dynamics are coupled with daily feeding−fasting cycle in part through BMAL1, which regulates the rhythmic expression levels of fission and mitophagy genes [74]. Mitochondrial morphology in cultured fibroblasts also displays circadian rhythmicity and is regulated through clock-dependent modification of the mitochondrial fission protein DRP1 [75]. Control of mitochondrial morphology by clock genes was also observed in livers from liver-specific *Bmal1* knockout mice [74] and from Clock^Δ^19 mice [76] as well as in mouse skeletal muscle and heart [77,78]. Mitochondrial dynamics involve massive changes in the organelles’ membranes. Indeed, lipidomics analyses performed on isolated mitochondria from mouse liver at different times of the day revealed that about a third of the mitochondrial lipidome exhibits daily rhythms [68]. Both the phase and the composition of the rhythmic lipids is dependent on feeding/fasting cycles and on the clock components PER1,2.

Of the approximately thousand different proteins that reside in mitochondria [79], most are encoded by the nuclear genome and imported into the mitochondria, while only 13 proteins are encoded by the mitochondrial DNA and are locally transcribed and synthesized [80]. The transcript levels of several nuclear-encoded mitochondrial proteins are altered in clock-deficient mice [78,81]. ChIP analysis also revealed that these genes’ promoters are occupied by BMAL1 [74,82,83]. Indeed, over a third of mitochondrial proteins accumulate in a daily manner and include key enzymes in carbohydrate metabolism, fatty acid uptake and oxidation, Krebs cycle, and the respiratory chain complexes [57,58,84].

Proteomic analysis of isolated mitochondria around the clock revealed that the rhythmic mitochondrial proteins mostly peak during the early light phase, while their respective transcripts are elevated in the beginning of the dark phase [84]. This suggests that posttranscriptional mechanisms, such as protein-translation [85,86] and various protein modifications (e.g., acetylation [87,88,89]), alongside the mitochondrial protein import machinery, potentially shape the phase of the rhythmic mitochondrial proteome [69]. In addition, a wide range of metabolites associated with mitochondrial metabolism display circadian rhythmicity, including ATP and NAD^+^ [75,87,90,91]. Such metabolic rhythms are likely to feed back to the clock function [92,93]. 

Respiration through oxidative phosphorylation is perhaps the most prominent role of mitochondria. Assays that measure OCR performed on cells and isolated mitochondria were used to uncover the circadian control of mitochondrial nutrient utilization and respiration. Circadian fluctuations in OCR measurements were observed in cultured cells, including C2C12 [87], HepG2 [94,95], and fibroblasts [96]. OCR measurements in primary hepatocytes isolated from mice in the beginning of the light and dark phase revealed higher respiration in the latter. This daily difference was diminished in hepatocytes isolated from liver-specific *Bmal1* knockout mice [74]. Experiments performed with mitochondria isolated from mouse liver around the clock provided further insight on mitochondrial nutrient utilization throughout the day [84]. In the presence of substrates such as palmitoyl + carnitine and palmitoyl-CoA + carnitine, mitochondria exhibit rhythmic respiration with zenith level early in the light phase, in accordance with elevated Carnitine palmitoyltransferase 1 (CPT1) protein levels. Carbohydrate (i.e., pyruvate and malate) utilization is rhythmic as well but peaks later during the light phase in line with Pyruvate dehydrogenase (PDH) accumulation. Remarkably, these daily rhythms in mitochondrial respiration are strongly influenced not only by the molecular circadian clock (i.e., *Per1,2* null mice), but also by nutrition type (e.g., high fat diet) and mealtime (i.e., nighttime restricted feeding).

Together, these studies suggest that mitochondrial biology exhibits daily rhythms, at least in mouse liver. It is conceivable that the observed temporal changes in mitochondrial fission-fusion, protein content, and the organelles’ function evolved in response to daily changes in nutrient availability and energetic demands.

### 2.3. Endoplasmic Reticulum (ER)

The ER is an elaborate network of lipid membranes that forms sites in which secretory and transmembrane proteins undergo folding and posttranslational modifications that are required for their function. Interconnected with the nuclear membrane as well as mitochondria, these organelles offer a range of functions for spatio-temporal investigations. 

As early as the 1970s and onwards, microscopic studies described diurnal changes in ER morphology that occur in rat hepatocytes [97,98]. Both the rough and smooth ER (with and without ribosomes, respectively) were proposed to vary in surface area and volume densities, presenting anti-phasic daily trends [98]. 

The ER constitutes the first step in the protein secretion pathway, which transports an estimated 30% of proteins, at least in humans [79,99]. In recent years, this process was proposed to oscillate throughout the day, based on around the clock mouse liver proteomics [57,58]. In these studies, the rhythmic proteome was enriched for secreted proteins with various functions: apolipoproteins, coagulation factors, complement factors, and serine protease inhibitors [57]. Furthermore, many components of the secretory pathway itself, such as ribosome docking, ER chaperones, ER, and Golgi vesicle-mediated transporters were found to be rhythmic [58]. This supports the assumption that regulation of protein secretion is rhythmic, yet functional assays are still lacking. Remarkably, similar to the rhythmic mitochondrial proteome detailed above, both studies note that the rhythmicity of many of these secretory proteins do not correspond to their transcript levels, suggesting a role for post-transcriptional mechanisms in control of their daily rhythmicity.

Collagen, the most abundant Extra Cellular Matrix (ECM) protein, is often thought to be found in constant levels [100,101]. Indeed, neither its transcript level nor its total protein levels are rhythmic; however, its synthesis, transport, and degradation are all circadian [100]. For instance, in mouse tendons, daily dynamics were recently detected across a range of different collagen fibrils, which differed in their peak times, thus explaining how the net effect is constant. In support of this, oscillations were observed in collagen secretion, transport, and degradation-related transcripts (e.g., *Sec61a2*, *Mia3*, *Pde4d,* and *Vps33b*), as well as their ribosome occupancy. Moreover, such collagen fiber assembly and disassembly maintain their circadian rhythmicity in a cell-autonomous manner in cultured fibroblasts. 

It is noteworthy that enrichment for the “protein folding” functional category was also reported in the daily rhythmic proteome [58]. These proteins’ expression was relatively aligned with their rhythmic transcripts. Protein folding in ER is crucial for cellular function and requires tight regulation. Following excessive accumulation of unfolded proteins, specific signaling pathways are activated, collectively termed Unfolded Protein Response (UPR) [102]. Treatment with the UPR-inducer tunicamycin at different times around the clock revealed that induction of several UPR components in mouse liver is dependent on the time of day [103]. Curiously, UPR-related genes undergo the rhythmic expression of a 12 h period (a harmonic of 24 h) with synchronized peak times. Phosphorylation of the UPR-related kinase IRE1α (Inositol-requiring transmembrane kinase/endoribonuclease 1α) and nuclear accumulation of the XBP1 (X-Box Binding Protein-1) transcription factor show corresponding rhythmicity. These rhythms persist in animals housed in constant dark and that are food-deprived, suggesting that they are endogenously driven [103]. In fact, XBP1 was recently further shown to regulate 12 h rhythmicity that goes beyond the UPR [104,105,106].

In other works, several mRNAs encoding for UPR signaling components were shown to oscillate in mouse liver with 24 h rhythmicity [107]. The UPR-activated transcription factor, ATF4 (Activating Transcription Factor 4), was shown to undergo circadian cycles, both in transcript and protein levels, both in mouse liver and cultured cells [108]. While *Clock* knockout animals show complete disruption of *Atf4* rhythms [108], *Cry1,2* double knockouts subjected to night-restricted feeding exhibit rhythmic activation of the pathway with a 24 h periodicity [103], highlighting the complex regulation of UPR.

As depicted above, several aspects of ER function, such as protein secretion and UPR, are rhythmic, and it would be interesting to examine in future studies the underlying molecular mechanisms as well as the functional implications.

### 2.4. Lysosomes

The lysosomal degradation system sequesters hydrolytic enzymes into membrane compartments, segregating their activity within the cell. Lysosomes, “digestive bodies” in Greek, were serendipitously discovered by biochemical fractionation [109,110] and soon after were identified in situ within cells by direct microscopy [111], which also revealed that they are morphologically heterogeneous among various cell types [112]. In the following years, it also became clear that lysosomes vary in their size and shape throughout the day, at least in rat heart and liver [98,113]. These rhythms do not come as a surprise, since lysosomes are highly influenced by feeding/fasting conditions. Starvation induces recycling and removal of unnecessary cytoplasmic components such as metabolites, proteins, lipids, and even other organelles. Accordingly, lysosome volume is larger during the light phase, namely when nocturnal rodents do not ingest food [113].

Lysosomes have received attention in chronobiology literature, somewhat indirectly, in relation to autophagy, a process in which they play a key role. Many aspects of autophagy are by now established as rhythmic. These include autophagy-related gene expression and protein accumulation, autophagic flux, as well as the appearance of autophagosomes [114,115]. The role of specific clock components in autophagy regulation has been examined. Interestingly, while an early study testing *Bmal1*-liver specific knockout mice showed that autophagy rhythmicity, and the overall autophagic response, is largely diminished [114], a recent study showed that whole body *Bmal1*-null mice present rhythmic autophagic flux similar to wild-type mice [115]. Future research is required to settle this apparent discrepancy. It is noteworthy that mitochondrial autophagy was found to be regulated by the circadian *Clock* gene in cardiac myocytes during ischemic stress [116]. In addition, REV-ERBα, AMPK, mTOR, and the autophagy-inducing kinase ULK1, which themselves exhibit circadian rhythmicity, were suggested to act as integrators of the nutrition−clocks−autophagy axis, as detailed in relevant reviews [117,118,119].

A case for circadian phagocytosis was recently made in a retinal-cell culture (ARPE-19 monolayers), which show rhythmic expression of phagocytosis-related genes, as well as lysosomal-associated membrane protein 1 (LAMP1) [120]. LAMP1, a lysosomal marker, is activated in these cells in a time-dependent manner, downstream to photoreceptor removal demands, demonstrating circadian regulation of lysosomal activation. 

Overall, different aspects of lysosomal biology show circadian rhythmicity from morphology to related gene expression, protein accumulation, as well as some relevant functions, yet the underlying molecular mechanisms in conjunction with the circadian clock are still lacking. 

## 3. Discussion

In his incessant wit, the world-renowned chronobiologist Michael Rosbash concludes his “50-year journey” in circadian genetics with: “The moral of the story is, as in real estate, ‘location, location, location.’” [121]. That is to say, in retrospect, his Nobel-prize-level success can be attributed to *location* in terms of where he performed his research, or his former student Paul Harding’s breakthrough approach in using fly heads instead of whole animals, and other fortuitous paths. To adopt and double-down on his sage advice, we discuss below the benefits of studying circadian rhythms in organelles. 

### 3.1. The Importance of Circadian Organelle Research

**Figure 2:**
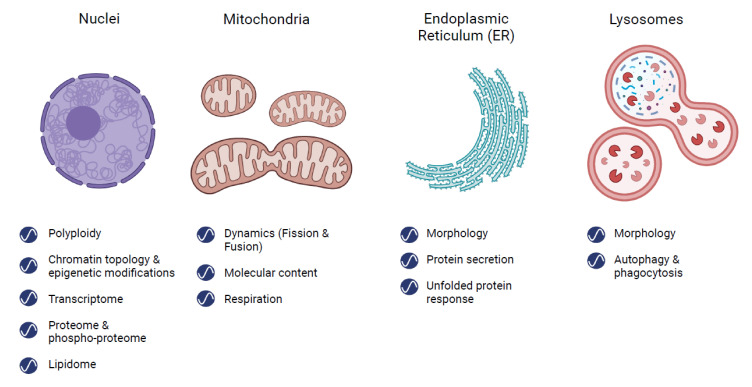
Circadian rhythmicity in subcellular organelles. Illustration of the major organelles discussed in this review: nuclei, mitochondria, endoplasmic reticulum, and lysosomes. Rhythmicity can be observed in the organelles’ structure and function, morphology, and key biochemical processes.

Much like different organs throughout the body, organelles have distinct functions, as were described throughout this review (Figure 2). Thus, investigating isolated parts can provide better resolution and deeper understanding of localized processes. For example, coverage of scarcely expressed proteins and transcription factors in whole cells is low, but they are more readily detected using purified nuclei. 

Detectability of the target of interest is of obvious importance for every study, regardless of the aspect of time. However, when addressing rhythmic properties, selecting the proper location becomes even more critical. Consider our group’s personal experience with circadian lipid research: in an earlier work [67], we utilized a lipidomics approach on mouse liver around the clock and detected rhythmicity in 17% of the lipids, the majority of them being triacylglycerols (TAGs). Interestingly, when we applied the same lipidomics technique on isolated nuclei and mitochondria, we saw a twofold increase in rhythmic lipids, but each organelle had opposing peak times of lipid molecules [68]. This demonstrates that, in practice, if a tissue contains anti-phasic rhythms in different organelles, these will be averaged-out when analyzing the tissue as a whole. Similar conclusions were drawn in respect to rhythmic protein levels, as described in [39].

### 3.2. The Challenges of Circadian Organelle Research

Despite the apparent advantages, studying isolated organelles brings about many challenges. While subcellular fractionation protocols are established (for example [122,123]), their purity is not always very high, and they might contain other organelle contaminants. Furthermore, a time-course experiment would require significant efforts in applying these additional steps around the clock and would likely require more biological material to start with (i.e., compared with straightforward sample collection). 

While of great interest and potential implications, much of the organelle-related rhythms known today and described herein are in fact based on descriptive methods and mostly rely on high-throughput omics techniques. Our knowledge regarding the functional significance of these changes is still lagging behind, predominantly because many of the relevant assays are demanding and difficult to perform around the clock. Furthermore, the presence of oscillations in isolated organelles may not be representative of their behavior within tissues or living organisms.

Furthermore, questions regarding the effect of rhythmicity in one organelle on another organelle are extremely challenging to perform as it is currently near impossible to directly interfere with a specific organelle in view of their indispensable roles. Having said that, there are some exceptions. For example, although mitochondria are considered as the cellular energy power plants, in cell culture, cells can perfectly grow and survive in the absence of mitochondrial DNA and consequently defective mitochondria (i.e., ρ^0^ cells), [124]. Hence, it would be interesting to examine the effect of mitochondria on rhythmicity of other cellular processes using these cells.

Despite some efforts to uncover the molecular mechanisms underlying different facets of organelle rhythmicity, relatively little is known. It is unclear to which extent these rhythms are driven by the molecular circadian clocks, how much is controlled at the transcriptional vs. the posttranscriptional levels, as well as within or outside the organelles. Obviously, this type of questions requires advanced expertise and methodologies both from the molecular and cell biology arenas.

### 3.3. Potential and Speculative Implications of Circadian Organelle Research

Importantly, the rhythmicity observed in different organelles and detailed above is most likely driven either directly or indirectly by the molecular circadian clock. Although currently there is no evidence to support this idea, one can speculate that organelles might harbor their own internal clock. Of particular interest are mitochondria, in view of their evolutionary shared past with prokaryotes, some of which harbor a circadian clock [125,126]. However, unlike cells that can be cultured for days to weeks without any intervention, to date it has been virtually impossible to keep isolated organelles viable in culture for several days. Such ex vivo “organelle-cultures” are extremely critical in order to determine organelle specific rhythmicity. Nonetheless, the notion of an organelle-intrinsic clock could be very appealing for exciting future research avenues relating to the hierarchy of the circadian system. Following the above-mentioned endeavor to find the clock’s location, and the historical transition in studying mammalian time-measuring capacity from whole organisms, to the SCN region of the brain, and subsequently to every cell in the body, adding another layer of circadian complexity, potentially in the form of “organelle-clocks”, seems to be a conceivable trajectory.

Following this narrative, irrespective of whether organelles harbor their own clock, it is likely that their circadian rhythmicity should be coordinated with the rhythmicity of other cellular compartments to optimize cellular functions, very similar to the observation that circadian rhythmicity of different organs in mammals is phase-coordinated through clock-dependent and independent mechanisms [127]. For example, temporal communication between organelles can be achieved through different metabolites, proteins, contact sites, as well as various signaling pathways. Based on the notion that the cell functions as a unit with internal logic and flow, it is tempting to speculate that oftentimes various molecular cargos can serve as time cues to support a sort of temporal organelle crosstalk. In this light, the observed temporal correlation between lipids in nuclei and mitochondria [68] could suggest that the same lipid species shuttle from one compartment to the other. Metabolites shuttling from mitochondria to the nucleus is known as “retrograde signaling” and is an important part of the cellular stress response, conveying information of metabolic status to the nucleus. One could conceive of a hypothetical cross-organelle cooperation in which stress response activates both mitochondria signaling to the nucleus and lysosomal autophagy to control organelle remodeling. Recently, a connection between retrograde signals and circadian clocks was shown in plants, with signals from mitochondria and chloroplasts affecting the plant clock system [128]. 

## 4. Summary

Technological advances and conceptual breakthroughs have occurred throughout the last decades in the fields of circadian and organelle biology. Growing evidence suggests that intracellular organelles are rhythmic, yet this line of research is still in its infancy. Many key questions regarding the functional significance and the underlying mechanisms remain open and pose exciting challenges for future molecular and cell biologists as they continue to push temporal and spatial boundaries.

## References

[1] Pittendrigh C.S. (1954). On temperature independence in the clock system controlling emergence time in drosophila. Proc. Natl. Acad. Sci. USA.

[2] Pittendrigh C.S. (1993). Temporal organization: Reflections of a darwinian clock-watcher. Annu. Rev. Physiol..

[3] Aviram R., Manella G. (2020). A metaphor that keeps on ticking: The ‘clock’ as a driving force in the history of chronobiology research. PTPBio.

[4] Moore R.Y., Eichler V.B. (1972). Loss of a circadian adrenal corticosterone rhythm following suprachiasmatic lesions in the rat. Brain Res..

[5] Stephan F.K., Zucker I. (1972). Circadian rhythms in drinking behavior and locomotor activity of rats are eliminated by hypothalamic lesions. Proc. Natl. Acad. Sci. USA.

[6] Yagita K., Tamanini F., Van der Horst G.T., Okamura H. (2001). Molecular mechanisms of the biological clock in cultured fibroblasts. Science.

[7] Yoo S.H., Yamazaki S., Lowrey P.L., Shimomura K., Ko C.H., Buhr E.D., Siepka S.M., Hong H.K., Oh W.J., Yoo O.J. (2004). PERIOD2::LUCIFERASE real-time reporting of circadian dynamics reveals persistent circadian oscillations in mouse peripheral tissues. Proc. Natl. Acad. Sci. USA.

[8] Nagoshi E., Saini C., Bauer C., Laroche T., Naef F., Schibler U. (2004). Circadian gene expression in individual fibroblasts: Cell-autonomous and self-sustained oscillators pass time to daughter cells. Cell.

[9] Balsalobre A., Marcacci L., Schibler U. (2000). Multiple signaling pathways elicit circadian gene expression in cultured Rat-1 fibroblasts. Curr. Biol..

[10] Swan J.A., Golden S.S., LiWang A., Partch C.L. (2018). Structure, function, and mechanism of the core circadian clock in cyanobacteria. J. Biol. Chem..

[11] Konopka R.J., Benzer S. (1971). Clock mutants of drosophila melanogaster. Proc. Natl. Acad. Sci. USA.

[12] Hardin P.E., Hall J.C., Rosbash M. (1990). Feedback of the Drosophila period gene product on circadian cycling of its messenger RNA levels. Nature.

[13] Bargiello T.A., Young M.W. (1984). Molecular genetics of a biological Clock in Drosophila. Proc. Natl. Acad. Sci. USA.

[14] Bargiello T.A., Jackson F.R., Young M.W. (1984). Restoration of circadian behavioural rhythms by gene transfer in Drosophila. Nature.

[15] Brown S.A., Kowalska E., Dallmann R. (2012). (Re)inventing the circadian feedback loop. Dev. Cell.

[16] Debruyne J.P., Noton E., Lambert C.M., Maywood E.S., Weaver D.R., Reppert S.M. (2006). A clock shock: Mouse clock is not required for circadian oscillator function. Neuron.

[17] Vitaterna M.H., King D.P., Chang A.M., Kornhauser J.M., Lowrey P.L., McDonald J.D., Dove W.F., Pinto L.H., Turek F.W., Takahashi J.S. (1994). Mutagenesis and mapping of a mouse gene, Clock, essential for circadian behavior. Science.

[18] Buhr E.D., Takahashi J.S., Kramer A., Merrow M. (2013). Molecular Components of the Mammalian Circadian Clock. Circadian Clocks. Handbook of Experi-mental Pharmacology.

[19] Dibner C., Schibler U., Albrecht U. (2010). The mammalian circadian timing system: Organization and coordination of central and peripheral clocks. Annu. Rev. Physiol..

[20] Honma S., Kawamoto T., Takagi Y., Fujimoto K., Sato F., Noshiro M., Kato Y., Honma K. (2002). Dec1 and Dec2 are regulators of the mammalian molecular clock. Nature.

[21] Kato Y., Kawamoto T., Fujimoto K., Noshiro M. (2014). Dec1/Stra13/Sharp2 and Dec2/Sharp1 coordinate physiological processes, including circadian rhythms in response to environmental stimuli. Curr. Top. Dev. Biol..

[22] Gachon F. (2007). Physiological Function of PARbZip circadian clock-controlled transcription factors. Ann. Med..

[23] Edgar R.S., Green E.W., Zhao Y., Van Ooijen G., Olmedo M., Qin X., Xu Y., Pan M., Valekunja U.K., Feeney K.A. (2012). Peroxiredoxins are conserved markers of circadian rhythms. Nature.

[24] O’Neill J.S., Reddy A.B. (2011). Circadian clocks in human red blood cells. Nature.

[25] Halberg F. (1959). Physiologic 24-hour periodicity; general and procedural considerations with reference to the adrenal cycle. Int. Z Vitam. Beih..

[26] Vitaterna M.H., Takahashi J.S., Turek F.W. (2001). Overview of circadian rhythms. Alcohol Res. Health.

[27] Dibner C. (2020). The importance of being rhythmic: Living in harmony with your body clocks. Acta Physiol..

[28] Schibler U., Gotic I., Saini C., Gos P., Curie T., Emmenegger Y., Sinturel F., Gosselin P., Gerber A., Fleury-Olela F. (2015). Clock-talk: Interactions between central and peripheral circadian oscillators in mammals. Cold Spring Harb. Symp. Quant. Biol..

[29] Balsalobre A., Brown S.A., Marcacci L., Tronche F., Kellendonk C., Reichardt H.M., Schutz G., Schibler U. (2000). Resetting of circadian time in peripheral tissues by glucocorticoid signaling. Science.

[30] Hirota T., Okano T., Kokame K., Shirotani-Ikejima H., Miyata T., Fukada Y. (2002). Glucose down-regulates *Per1* and *Per2*mRNA levels and induces circadian gene expression in cultured Rat-1 fibroblasts. J. Biol. Chem..

[31] Yamajuku D., Inagaki T., Haruma T., Okubo S., Kataoka Y., Kobayashi S., Ikegami K., Laurent T., Kojima T., Noutomi K. (2012). Real-time monitoring in three-dimensional hepatocytes reveals that insulin acts as a synchronizer for liver clock. Sci. Rep..

[32] Adamovich Y., Ladeuix B., Golik M., Koeners M.P., Asher G. (2017). Rhythmic oxygen levels reset circadian clocks through Hif1α. Cell Metab..

[33] Adamovich Y., Ladeuix B., Sobel J., Manella G., Neufeld-Cohen A., Assadi M.H., Golik M., Kuperman Y., Tarasiuk A., Koeners M.P. (2019). Oxygen and carbon dioxide rhythms are circadian clock controlled and differentially directed by behavioral signals. Cell Metab..

[34] Uchiyama Y., Saito K. (1982). A morphometric study of 24-hour variations in subcellular structures of the rat pancreatic acinar cell. Cell Tissue Res..

[35] Uchiyama Y., Asari A. (1984). A morphometric study of the variations in subcellular structures of rat hepatocytes during 24 hours. Cell Tissue Res..

[36] Aviram R., Manella G., Asher G. (2021). The liver by day and by night. J. Hepatol..

[37] Gentric G., Celton-Morizur S., Desdouets C. (2012). Polyploidy and liver proliferation. Clin. Res. Hepatol. Gastroenterol..

[38] Duncan A.W. (2013). Aneuploidy, polyploidy and ploidy reversal in the liver. Semin. Cell Dev. Biol..

[39] Wang J., Mauvoisin D., Martin E., Atger F., Galindo A.N., Dayon L., Sizzano F., Palini A., Kussmann M., Waridel P. (2017). Nuclear proteomics uncovers diurnal regulatory landscapes in mouse liver. Cell Metab..

[40] Chao H.W., Doi M., Fustin J.M., Chen H., Murase K., Maeda Y., Hayashi H., Tanaka R., Sugawa M., Mizukuchi N. (2017). Circadian clock regulates hepatic polyploidy by modulating Mkp1-Erk1/2 signaling pathway. Nat. Commun..

[41] Kim Y.H., Lazar M.A. (2020). Transcriptional control of circadian rhythms and metabolism: A matter of time and space. Endocr. Rev..

[42] Yeung J., Naef F. (2018). Rhythms of the genome: Circadian dynamics from chromatin topology, tissue-specific gene expression, to behavior. Trends Genet..

[43] Aguilar-Arnal L., Hakim O., Patel V.R., Baldi P., Hager G.L., Sassone-Corsi P. (2013). Cycles in spatial and temporal chromosomal organization driven by the circadian clock. Nat. Struct. Mol. Biol..

[44] Zhao H., Sifakis E.G., Sumida N., Millan-Arino L., Scholz B.A., Svensson J.P., Chen X., Ronnegren A.L., Mallet de Lima C.D., Varnoosfaderani F.S. (2015). PARP1-and CTCF-mediated interactions between active and repressed chromatin at the lamina promote oscillating transcription. Mol. Cell.

[45] Asher G., Reinke H., Altmeyer M., Gutierrez-Arcelus M., Hottiger M.O., Schibler U. (2010). Poly (ADP-ribose) polymerase 1 participates in the phase entrainment of circadian clocks to feeding. Cell.

[46] Lin S.T., Zhang L., Lin X., Zhang L.C., Garcia V.E., Tsai C.W., Ptacek L., Fu Y.H. (2014). Nuclear envelope protein man1 regulates clock through Bmal1. eLife.

[47] Bu B., He W., Song L., Zhang L. (2019). Nuclear envelope protein Man1 regulates the drosophila circadian clock via period. Neurosci. Bull..

[48] Furlan-Magaril M., Ando-Kuri M., Arzate-Mejia R.G., Morf J., Cairns J., Roman-Figueroa A., Tenorio-Hernandez L., Poot-Hernandez A.C., Andrews S., Varnai C. (2021). The global and promoter-centric 3d genome organization temporally resolved during a circadian cycle. Genome Biol..

[49] Brunet A., Forsberg F., Fan Q., Saether T., Collas P. (2019). Nuclear lamin B1 interactions with chromatin during the circadian cycle are uncoupled from periodic gene expression. Front. Genet..

[50] Pacheco-Bernal I., Becerril-Perez F., Aguilar-Arnal L. (2019). Circadian rhythms in the three-dimensional genome: Implications of chromatin interactions for cyclic transcription. Clin. Epigenetics.

[51] Kim Y.H., Marhon S.A., Zhang Y., Steger D.J., Won K.J., Lazar M.A. (2018). Rev-erbα dynamically modulates chromatin looping to control circadian gene transcription. Science.

[52] Mermet J., Yeung J., Hurni C., Mauvoisin D., Gustafson K., Jouffe C., Nicolas D., Emmenegger Y., Gobet C., Franken P. (2018). Clock-dependent chromatin topology modulates circadian transcription and behavior. Genes Dev..

[53] Mermet J., Yeung J., Naef F. (2021). Oscillating and stable genome topologies underlie hepatic physiological rhythms during the circadian cycle. PLoS Genet..

[54] Zhang R., Lahens N.F., Ballance H.I., Hughes M.E., Hogenesch J.B. (2014). A circadian gene expression atlas in mammals: Implications for biology and medicine. Proc. Natl. Acad. Sci. USA.

[55] Doherty C.J., Kay S.A. (2010). Circadian control of global gene expression patterns. Annu. Rev. Genet..

[56] Torres M., Becquet D., Franc J.L., Francois-Bellan A.M. (2018). Circadian processes in the RNA life cycle. Wiley Interdiscip. Rev. RNA.

[57] Mauvoisin D., Wang J., Jouffe C., Martin E., Atger F., Waridel P., Quadroni M., Gachon F., Naef F. (2014). Circadian clock-dependent and-independent rhythmic proteomes implement distinct diurnal functions in mouse liver. Proc. Natl. Acad. Sci. USA.

[58] Robles M.S., Cox J., Mann M. (2014). In-Vivo quantitative proteomics reveals a key contribution of post-transcriptional mechanisms to the circadian regulation of liver metabolism. PLoS Genet..

[59] Robles M.S., Humphrey S.J., Mann M. (2017). Phosphorylation is a central mechanism for circadian control of metabolism and physiology. Cell Metab..

[60] Wang Y., Song L., Liu M., Ge R., Zhou Q., Liu W., Li R., Qie J., Zhen B., Wang Y. (2018). A proteomics landscape of circadian clock in mouse liver. Nat. Commun..

[61] Korge S., Maier B., Bruning F., Ehrhardt L., Korte T., Mann M., Herrmann A., Robles M.S., Kramer A. (2018). The non-classical nuclear import carrier transportin 1 modulates circadian rhythms through its effect on PER1 nuclear localization. PLoS Genet..

[62] Lee Y., Shen Y., Francey L.J., Ramanathan C., Sehgal A., Liu A.C., Hogenesch J.B. (2019). The NRON complex controls circadian clock function through regulated per and CRY nuclear translocation. Sci. Rep..

[63] Lee Y., Jang A.R., Francey L.J., Sehgal A., Hogenesch J.B. (2015). KPNB1 mediates PER/CRY nuclear translocation and circadian clock function. eLife.

[64] Chua E.C., Shui G., Lee I.T., Lau P., Tan L.C., Yeo S.C., Lam B.D., Bulchand S., Summers S.A., Puvanendran K. (2013). Extensive diversity in circadian regulation of plasma lipids and evidence for different circadian metabolic phenotypes in humans. Proc. Natl. Acad. Sci. USA.

[65] Kasukawa T., Sugimoto M., Hida A., Minami Y., Mori M., Honma S., Honma K., Mishima K., Soga T., Ueda H.R. (2012). Human blood metabolite timetable indicates internal body time. Proc. Natl. Acad. Sci. USA.

[66] Loizides-Mangold U., Perrin L., Vandereycken B., Betts J.A., Walhin J.P., Templeman I., Chanon S., Weger B.D., Durand C., Robert M. (2017). Lipidomics reveals diurnal lipid oscillations in human skeletal muscle persisting in cellular myotubes cultured in vitro. Proc. Natl. Acad. Sci. USA.

[67] Adamovich Y., Rousso-Noori L., Zwighaft Z., Neufeld-Cohen A., Golik M., Kraut-Cohen J., Wang M., Han X., Asher G. (2014). Circadian clocks and feeding time regulate the oscillations and levels of hepatic triglycerides. Cell Metab..

[68] Aviram R., Manella G., Kopelman N., Neufeld-Cohen A., Zwighaft Z., Elimelech M., Adamovich Y., Golik M., Wang C., Han X. (2016). Lipidomics analyses reveal temporal and spatial lipid organization and uncover daily oscillations in intracellular organelles. Mol. Cell.

[69] Manella G., Asher G. (2016). The circadian nature of mitochondrial biology. Front. Endocrinol..

[70] Sardon Puig L., Valera-Alberni M., Canto C., Pillon N.J. (2018). Circadian rhythms and mitochondria: Connecting the dots. Front. Genet..

[71] Wai T., Langer T. (2016). Mitochondrial dynamics and metabolic regulation. Trends Endocrinol. Metab..

[72] Liesa M., Shirihai O.S. (2013). Mitochondrial dynamics in the regulation of nutrient utilization and energy expenditure. Cell Metab..

[73] Uchiyama Y. (1981). Circadian alterations in tubular structures on the outer mitochondrial membrane of rat hepatocytes. Cell Tissue Res..

[74] Jacobi D., Liu S., Burkewitz K., Kory N., Knudsen N.H., Alexander R.K., Unluturk U., Li X., Kong X., Hyde A.L. (2015). Hepatic Bmal1 regulates rhythmic mitochondrial dynamics and promotes metabolic fitness. Cell Metab..

[75] Schmitt K., Grimm A., Dallmann R., Oettinghaus B., Restelli L.M., Witzig M., Ishihara N., Mihara K., Ripperger J.A., Albrecht U. (2018). Circadian control of DRP1 activity regulates mitochondrial dynamics and bioenergetics. Cell Metab..

[76] Xu L., Cheng Q., Hua B., Cai T., Lin J., Yuan G., Yan Z., Li X., Sun N., Lu C. (2018). Circadian gene clock regulates mitochondrial morphology and functions by posttranscriptional way. bioRxiv.

[77] Andrews J.L., Zhang X., McCarthy J.J., McDearmon E.L., Hornberger T.A., Russell B., Campbell K.S., Arbogast S., Reid M.B., Walker J.R. (2010). Clock and BMAL1 Regulate MyoD and are necessary for maintenance of skeletal muscle phenotype and function. Proc. Natl. Acad. Sci. USA.

[78] Kohsaka A., Das P., Hashimoto I., Nakao T., Deguchi Y., Gouraud S.S., Waki H., Muragaki Y., Maeda M. (2014). The circadian clock maintains cardiac function by regulating mitochondrial metabolism in mice. PLoS ONE.

[79] Thul P.J., Akesson L., Wiking M., Mahdessian D., Geladaki A., Blal H.A., Alm T., Asplund A., Bjork L., Breckels L.M. (2017). A subcellular map of the human proteome. Science.

[80] Roger A.J., Munoz-Gomez S.A., Kamikawa R. (2017). The origin and diversification of mitochondria. Curr Biol..

[81] Gong C., Li C., Qi X., Song Z., Wu J., Hughes M.E., Li X. (2015). The daily rhythms of mitochondrial gene expression and oxidative stress regulation are altered by aging in the mouse liver. Chronobiol. Int..

[82] Koike N., Yoo S.H., Huang H.C., Kumar V., Lee C., Kim T.K., Takahashi J.S. (2012). Transcriptional architecture and chromatin landscape of the core circadian clock in mammals. Science.

[83] Rey G., Cesbron F., Rougemont J., Reinke H., Brunner M., Naef F. (2011). Genome-wide and phase-specific DNA-binding rhythms of BMAL1 control circadian output functions in mouse liver. PLoS Biol..

[84] Neufeld-Cohen A., Robles M.S., Aviram R., Manella G., Adamovich Y., Ladeuix B., Nir D., Rousso-Noori L., Kuperman Y., Golik M. (2016). Circadian control of oscillations in mitochondrial rate-limiting enzymes and nutrient utilization by period proteins. Proc. Natl. Acad. Sci. USA.

[85] Atger F., Gobet C., Marquis J., Martin E., Wang J., Weger B., Lefebvre G., Descombes P., Naef F., Gachon F. (2015). Circadian and feeding rhythms differentially affect rhythmic mRNA transcription and translation in mouse liver. Proc. Natl. Acad. Sci. USA.

[86] Janich P., Arpat A.B., Castelo-Szekely V., Lopes M., Gatfield D. (2015). Ribosome profiling reveals the rhythmic liver translatome and circadian clock regulation by upstream open reading frames. Genome Res..

[87] Peek C.B., Affinati A.H., Ramsey K.M., Kuo H.Y., Yu W., Sena L.A., Ilkayeva O., Marcheva B., Kobayashi Y., Omura C. (2013). Circadian clock NAD+ cycle drives mitochondrial oxidative metabolism in mice. Science.

[88] Mauvoisin D., Atger F., Dayon L., Nunez Galindo A., Wang J., Martin E., Da Silva L., Montoliu I., Collino S., Martin F.P. (2017). Circadian and feeding rhythms orchestrate the diurnal liver acetylome. Cell Rep..

[89] Masri S., Patel V.R., Eckel-Mahan K.L., Peleg S., Forne I., Ladurner A.G., Baldi P., Imhof A., Sassone-Corsi P. (2013). Circadian acetylome reveals regulation of mitochondrial metabolic pathways. Proc. Natl. Acad. Sci. USA.

[90] Ramsey K.M., Yoshino J., Brace C.S., Abrassart D., Kobayashi Y., Marcheva B., Hong H.K., Chong J.L., Buhr E.D., Lee C. (2009). Circadian clock feedback cycle through NAMPT-mediated NAD+ biosynthesis. Science.

[91] Nakahata Y., Sahar S., Astarita G., Kaluzova M., Sassone-Corsi P. (2009). Circadian control of the NAD+ salvage pathway by CLOCK-SIRT1. Science.

[92] Reinke H., Asher G. (2019). Crosstalk between metabolism and circadian clocks. Nat. Rev. Mol. Cell Biol..

[93] Asher G., Sassone-Corsi P. (2015). Time for food: The intimate interplay between nutrition, metabolism, and the circadian clock. Cell.

[94] Cela O., Scrima R., Pazienza V., Merla G., Benegiamo G., Augello B., Fugetto S., Menga M., Rubino R., Fuhr L. (2016). Clock genes-dependent acetylation of complex I sets rhythmic activity of mitochondrial OxPhos. Biochim. Biophys. Acta Mol. Cell Res..

[95] Scrima R., Cela O., Merla G., Augello B., Rubino R., Quarato G., Fugetto S., Menga M., Fuhr L., Relogio A. (2016). Clock-genes and mitochondrial respiratory activity: Evidence of a reciprocal interplay. Biochim. Biophys. Acta Mol. Cell Res..

[96] Pacelli C., Rotundo G., Lecce L., Menga M., Bidollari E., Scrima R., Cela O., Piccoli C., Cocco T., Vescovi A.L. (2019). Parkin mutation affects clock gene-dependent energy metabolism. Int. J. Mol. Sci..

[97] Chedid A., Nair V. (1972). diurnal rhythm in endoplasmic reticulum of rat liver: Electron microscopic study. Science.

[98] Uchiyama Y. (1990). Rhythms in morphology and function of hepatocytes. J. Gastroenterol. Hepatol..

[99] Braakman I., Bulleid N.J. (2011). Protein folding and modification in the mammalian endoplasmic reticulum. Annu. Rev. Biochem..

[100] Chang J., Garva R., Pickard A., Yeung C.C., Mallikarjun V., Swift J., Holmes D.F., Calverley B., Lu Y., Adamson A. (2020). Circadian control of the secretory pathway maintains collagen homeostasis. Nat. Cell Biol..

[101] Heinemeier K.M., Schjerling P., Heinemeier J., Magnusson S.P., Kjaer M. (2013). Lack of tissue renewal in human adult achilles tendon is revealed by nuclear bomb (14) C. FASEB J..

[102] Hetz C., Papa F.R. (2018). The unfolded protein response and cell fate control. Mol. Cell.

[103] Cretenet G., Le Clech M., Gachon F. (2010). Circadian clock-coordinated 12 hr period rhythmic activation of the IRE1α pathway controls lipid metabolism in mouse liver. Cell Metab..

[104] Pan Y., Ballance H., Meng H., Gonzalez N., Kim S.M., Abdurehman L., York B., Chen X., Schnytzer Y., Levy O. (2020). 12-h clock regulation of genetic information flow by XBP1s. PLoS Biol..

[105] Meng H., Gonzales N.M., Lonard D.M., Putluri N., Zhu B., Dacso C.C., York B., O’Malley B.W. (2020). XBP1 links the 12-hour clock to nafld and regulation of membrane fluidity and lipid homeostasis. Nat. Commun..

[106] Zhu B., Zhang Q., Pan Y., Mace E.M., York B., Antoulas A.C., Dacso C.C., O’Malley B.W. (2017). A cell-autonomous mammalian 12 hr clock coordinates metabolic and stress rhythms. Cell Metab..

[107] Bu Y., Yoshida A., Chitnis N., Altman B.J., Tameire F., Oran A., Gennaro V., Armeson K.E., McMahon S.B., Wertheim G.B. (2018). A PERK-miR-211 axis suppresses circadian regulators and protein synthesis to promote cancer cell survival. Nat. Cell Biol..

[108] Koyanagi S., Hamdan A.M., Horiguchi M., Kusunose N., Okamoto A., Matsunaga N., Ohdo S. (2011). cAMP-response element (CRE)-mediated transcription by activating transcription factor-4 (ATF4) is essential for circadian expression of the Period2 gene. J. Biol. Chem..

[109] De Duve C., Pressman B.C., Gianetto R., Wattiaux R., Appelmans F. (1955). Tissue fractionation studies. 6. intracellular distribution patterns of enzymes in rat-liver tissue. Biochem. J..

[110] De Duve C. (2005). The lysosome turns fifty. Nat. Cell Biol..

[111] Novikoff A.B., Beaufay H., De Duve C. (1956). Electron microscopy of lysosomerich fractions from rat liver. J. Biophys. Biochem. Cytol..

[112] Ohsumi Y. (2014). Historical Landmarks of autophagy research. Cell Res..

[113] Pfeifer U., Strauss P. (1981). Autophagic vacuoles in heart muscle and liver. a comparative morphometric study including circadian variations in meal-fed rats. J. Mol. Cell Cardiol..

[114] Ma D., Panda S., Lin J.D. (2011). Temporal orchestration of circadian autophagy rhythm by C/EBPβ. EMBO J..

[115] Ryzhikov M., Ehlers A., Steinberg D., Xie W., Oberlander E., Brown S., Gilmore P.E., Townsend R.R., Lane W.S., Dolinay T. (2019). Diurnal rhythms spatially and temporally organize autophagy. Cell Rep..

[116] Rabinovich-Nikitin I., Rasouli M., Reitz C.J., Posen I., Margulets V., Dhingra R., Khatua T.N., Thliveris J.A., Martino T.A., Kirshenbaum L.A. (2021). Mitochondrial autophagy and cell survival is regulated by the circadian clock gene in cardiac myocytes during ischemic stress. Autophagy.

[117] Ma D., Li S., Molusky M.M., Lin J.D. (2012). Circadian autophagy rhythm: A link between clock and metabolism?. Trends Endocrinol. Metab..

[118] Panda S. (2016). Circadian physiology of metabolism. Science.

[119] Wang X., Xu Z., Cai Y., Zeng S., Peng B., Ren X., Yan Y., Gong Z. (2020). Rheostatic balance of circadian rhythm and autophagy in metabolism and disease. Front. Cell Dev. Biol..

[120] Milicevic N., Mazzaro N., De Bruin I., Wils E., Ten Brink J., Asbroek A.T., Mendoza J., Bergen A., Felder-Schmittbuhl M.P. (2019). Rev-Erbα and photoreceptor outer segments modulate the circadian clock in retinal pigment epithelial cells. Sci. Rep..

[121] Rosbash M. (2017). A 50-year personal journey: Location, gene expression, and circadian rhythms. Cold Spring Harb. Perspect. Biol..

[122] Aviram R., Wang C., Han X., Asher G. (2021). A lipidomics view of circadian biology. Methods Mol. Biol..

[123] Radhakrishnan A., Goldstein J.L., McDonald J.G., Brown M.S. (2008). Switch-like control of SREBP-2 transport triggered by small changes in ER cholesterol: A delicate balance. Cell Metab..

[124] King M.P., Attardi G. (1989). Human cells lacking mtDNA: Repopulation with exogenous mitochondria by complementation. Science.

[125] Eelderink-Chen Z., Bosman J., Sartor F., Dodd A.N., Kovacs A.T., Merrow M. (2021). A circadian clock in a nonphotosynthetic prokaryote. Sci. Adv..

[126] Cohen S.E., Golden S.S. (2015). Circadian rhythms in cyanobacteria. Microbiol. Mol. Biol. Rev..

[127] Manella G., Sabath E., Aviram R., Dandavate V., Ezagouri S., Golik M., Adamovich Y., Asher G. (2021). The liver-clock coordinates rhythmicity of peripheral tissues in response to feeding. Nat. Metab..

[128] Jones M.A. (2019). Retrograde signalling as an informant of circadian timing. New Phytol..

